# Spatial blurring in laser speckle imaging in inhomogeneous turbid media

**DOI:** 10.1038/s41598-017-17010-z

**Published:** 2017-12-04

**Authors:** Luka Vitomir, Joris Sprakel, Jasper van der Gucht

**Affiliations:** Wageningen University and Research Center, Department of Physical Chemistry and Soft Matter, Wageningen, 6708 WE Netherlands

## Abstract

Laser speckle imaging (LSI) has developed into a versatile tool to image dynamical processes in turbid media, such as subcutaneous blood perfusion and heterogeneous dynamics in soft materials. Spatially resolved information about local dynamics is obtained by measuring time-dependent correlation functions of multiply scattered light. Due to the diffusive nature of photons in highly scattering media, the measured signal is a convolution of the local dynamics in the material and the spatial distribution of photons. This spatial averaging inevitably leads to a loss of resolution, which must be taken into account for a correct interpretation of LSI measurements. In this paper we derive analytical expressions to quantify the effects of spatial blurring in backscatter LSI for materials with heterogeneous dynamics. Using the diffusion approximation, we calculate the photon density distribution for a semi-infinite material, and we predict the effect of dynamic heterogeneity on the measured correlation function. We verify our theoretical expressions using random walk simulations. Our results show that LSI measurements in dynamically heterogeneous materials should be interpreted with caution, especially when only a single wavelength and correlation time are used to obtain the dynamical map.

## Introduction

Many disordered materials, such as rubbers, plastics, foams, suspensions, and biological tissue strongly scatter light, which makes a direct observation of structure and dynamics, for example with light microscopy, very difficult. However, detailed information about the dynamical processes in such non-transparent materials can be obtained by analyzing the temporal fluctuations of light that is multiply scattered in the material. This forms the basis of diffusing-wave spectroscopy (DWS)^1–3^, a powerful technique to study Brownian motion or flow in concentrated colloidal systems^4–6^ or to extract viscoelastic moduli of soft materials from the dynamics of embedded tracer particles^7^. While DWS has been applied mostly to uniform samples, it can also be used as an imaging tool for characterizing spatially resolved dynamics in heterogeneous turbid media^8–10^. A particularly useful realization of this is laser speckle imaging (LSI). In LSI, the sample is illuminated with a plane wave of coherent light. Photons that enter the sample undergo many scattering events before leaving the sample again and reaching the camera. Each camera pixel receives many photons that have scattered from different positions in the sample; the resulting path length differences create a random interference pattern that is known as a speckle pattern. Movement of the scattering particles causes temporal fluctuations in the speckle pattern, which can be quantified by calculating for each speckle the intensity autocorrelation function *g*_2_(*τ*) = 〈*I*(0)*I*(*τ*)〉/〈*I*〉^2^, with *I*(*τ*) the intensity at time *τ*. The decay rate of this autocorrelation function is directly related to the dynamics of the scatterers in the material. Because the photons that reach the detector have scattered many times inside the sample, decorrelation already occurs when the scatterers have moved only a fraction of the wavelength. This makes it possible to measure particle displacements as small as a few nanometers with LSI^1^.

Because of its high sensitivity, its relative cost-effectiveness, and its non-invasive nature, LSI has become an attractive tool in bio-medical imaging^11–16^ and materials science^17–22^. However, obtaining quantitative information from LSI measurements remains difficult, because the decay of the autocorrelation function depends not only on the internal dynamics of the material, but also on the distribution of photon paths through the sample. This means that an accurate model is needed for the propagation of photons in the turbid medium. Usually, it is assumed that the propagation of light in the material can be approximated as a diffusion process, so that the path followed by an individual photon can be described as a random walk^23–25^. The distribution of path lengths can then be calculated by solving the diffusion equation. For samples in which the particle dynamics does not depend on position, this yields a direct relation between the intensity autocorrelation function and the mean-square displacement of the particles 〈Δ*r* ^2^(*τ*)〉^2,4^. However, for dynamically inhomogeneous samples, the information carried by the speckle pattern depends on which regions the photons have probed. Since each photon takes a different path through the sample, LSI measurements performed on inhomogeneous samples actually represent averages, resulting from a convolution of dynamic processes in the sample and the spatial probability distribution of photons in the sample^26,27^. This leads to spatial blurring and loss of resolution and thus limits the ability of LSI to detect dynamic heterogeneities^28^. For a correct interpretation of LSI measurements on dynamically heterogeneous samples, it is therefore necessary to quantify the effects of spatial averaging.

The typical distance travelled by photons before leaving the sample in a backscatter experiment is a few times the transport mean free path *l* ^*^^20^, which is the average step length of the photon random walk. One would therefore expect the penetration depth of light into the sample and the extent of spatial blurring to be also a few times *l* ^*^^29,30^. However, it is clear that the effect of blurring must also depend strongly on the correlation time *τ*, since the initial decay of the correlation function (at small *τ*) is much more sensitive to long photon paths than the long-time decay^1^. Hence, to fully appreciate how spatial averaging affects the measured LSI signal at different time scales, a more detailed calculation, taking all possible photon paths into account, is needed. Such an analysis has been performed for a multilayer medium, in which the sample is heterogeneous only in the *z*-direction (perpendicular to the surface)^10,31,32^. Here, we extend these findings to dynamically heterogeneous materials with arbitrary spatial distributions of particle dynamics. Using the diffusion approximation, we calculate the photon density distribution in the material and we derive an analytical expression that relates the autocorrelation function measured in a particular location to the distribution of mean-square displacements in the material. We then use this result to evaluate the lateral resolution of LSI, and we show how this depends on the correlation time. We verify our results using random walk simulations.

## LSI theory

**Figure 1:**
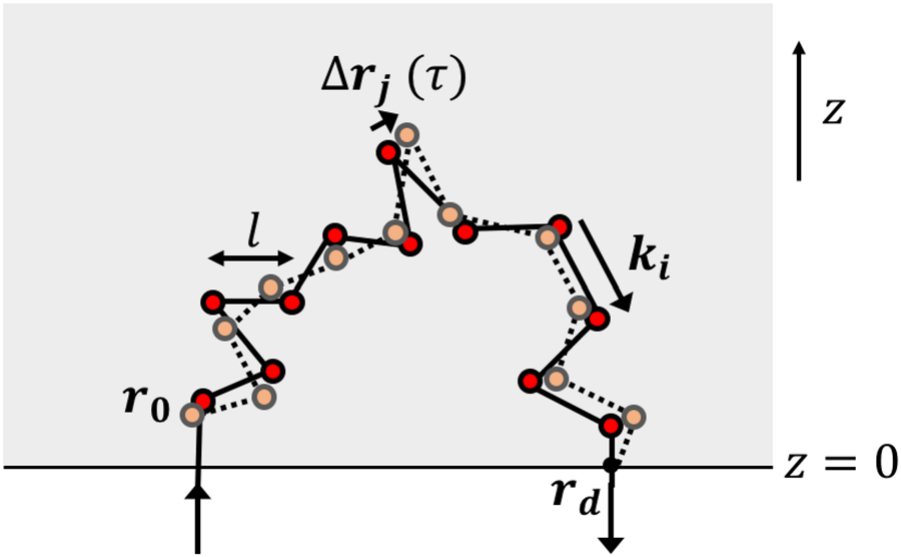
A photon trajectory: after entering the sample, a photon performs a random walk with mean free path *l* through the material before exiting the sample at ***r***_***d***_. The signal detected at ***r***_***d***_ is the result of all the random walks ending at ***r***_***d***_. The random walk is assumed to start at the first scattering event, ***r***_**0**_. Movement of the particles in a time *τ* leads to a change in the phase of the light wave, Δ*ϕ*_*p*_(*τ*) = ∑_*i*_ ***q***_***i***_ ⋅ Δ***r***_***i***_(*τ*) with ***q***_***i***_ = ***k***_***i***_ − ***k***_*i*−1_ the scattering vector transfer of scattering event *i*.

We consider LSI in the backscattering geometry for samples that are much thicker than the penetration depth of light in the material, so that the material can be considered as a semi-infinite half-space. We assume that no photon absorption takes place. Photons enter the material at a particular location ***r***_**0**_ and then undergo a sequence of scattering events before leaving the sample again at a point ***r***_***d***_ where they are detected by a camera (Fig. 1). The transport of photons in the material is characterized by the mean free path *l*, which is the average distance between two scattering events; it depends on the number density *ρ* and the scattering cross-section *σ* of the particles, *l* = 1/*ρσ*. The transport mean free path *l* ^*^ is the distance over which the direction of light becomes randomized; it is related to the mean free path by *l* ^*^ = *l*/〈1 − *cosθ*〉, where *θ* is the scattering angle and where the average is taken over the scattering form factor of the particle. For very small particles the scattering is isotropic and *l* ^*^ ≈ *l*, while for larger particles the scattering is peaked in the forward direction so that *l* ^*^ > *l*. The transport mean free path can be determined experimentally^33,34^ or it can be calculated from Mie theory^23^. In the Rayleigh limit, for particles that are small compared to the wavelength of light, *l* ^*^ can be several tens to hundreds of micrometers large and increases very strongly with increasing wavelength, as ${l}^{\ast }\sim {\lambda }^{4}$.

When the total path length of a photon is much larger than *l* ^*^, the photon trajectory can be described as a random walk with average step size *l* ^*^. The total electric field collected at a particular location ***r***_***d***_ is the superposition of the fields of all photon paths *p* ending at ***r***_***d***_:

1 $$E({{\boldsymbol{r}}}_{{\boldsymbol{d}}},t)=\sum _{p}{E}_{p}\exp (i{\varphi }_{p}(t))$$

where *E*_*p*_ is the amplitude of the electric field from path *p* and *ϕ*_*p*_(*t*) its phase at time *t*. The electric field correlation function *g*_1_(*τ*), which is related to the experimentally measured intensity correlation function *g*_2_(*τ*) by the Siegert relation, *g*_2_(*τ*) = 1 + *βg*_1_(*τ*)^2^ with *β* an experimental constant of order unity, can then be written as

2 $$\begin{array}{rcl}{g}_{1}({{\boldsymbol{r}}}_{{\boldsymbol{d}}},\tau ) & = & \frac{\langle E({{\boldsymbol{r}}}_{{\boldsymbol{d}}},\mathrm{0)}{E}^{\ast }({{\boldsymbol{r}}}_{{\boldsymbol{d}}},\tau )\rangle }{|E({{\boldsymbol{r}}}_{{\boldsymbol{d}}}{)|}^{2}}\\  & = & \frac{1}{|E({{\boldsymbol{r}}}_{{\boldsymbol{d}}}{)|}^{2}}\sum _{p,p^{\prime} }{E}_{p}{E}_{p^{\prime} }\langle \exp (i[{\varphi }_{p}(\tau )-{\varphi }_{p^{\prime} }\mathrm{(0)}])\rangle \\  & = & \sum _{p}{P}_{p}({{\boldsymbol{r}}}_{{\boldsymbol{d}}})\langle \exp (i{\rm{\Delta }}{\varphi }_{p}(\tau ))\rangle \end{array}$$

where ${P}_{p}({{\boldsymbol{r}}}_{{\boldsymbol{d}}})={E}_{p}^{2}/|E({{\boldsymbol{r}}}_{{\boldsymbol{d}}}{)|}^{2}={I}_{p}/I({{\boldsymbol{r}}}_{{\boldsymbol{d}}})$ is the fraction of the scattered intensity at ***r***_***d***_ coming from path *p*. Terms with *p* ≠ *p*′ do not contribute, because light waves belonging to different paths are uncorrelated. The phase shift Δ*ϕ*_*p*_(*τ*) = *ϕ*_*p*_(*τ*) − *ϕ*_*p*_(0) is due to movement of the *N*_*p*_ scatterers along path *p* and can be written as^1,4^

3 $${\rm{\Delta }}{{\varphi }}_{p}(\tau )=\sum _{i=1}^{{N}_{p}}{{\boldsymbol{q}}}_{{\boldsymbol{i}}}\cdot {\rm{\Delta }}{{\boldsymbol{r}}}_{{\boldsymbol{i}}}(\tau )$$

where ***q***_***i***_ = ***k***_***i***_ − ***k***_***i***−**1**_ is the wave vector transfer of scattering event *i*, with a magnitude that is determined by the scattering angle as *q* = 2*k*_0_sin(*θ*/2), and with Δ***r***_***i***_(*τ*) = ***r***_***i***_(*τ*) − ***r***_***i***_(0) the displacement of scatterer *i* in the time interval *τ*. For Brownian motion, the Δ***r***_***i***_(*τ*) are random Gaussian variables and the average in Eq. (2) is readily obtained^1^ to give:

4 $${g}_{1}(\tau )=\sum _{p}{P}_{p}({{\boldsymbol{r}}}_{{\boldsymbol{d}}})\exp (-\tfrac{1}{6}\langle {q}^{2}\rangle \sum _{i=1}^{{N}_{p}}\langle {\rm{\Delta }}{r}_{i}{(\tau )}^{2}\rangle )$$

with $\langle {q}^{2}\rangle =2{k}_{0}^{2}\langle 1-\,\cos \,\theta \rangle =2{k}_{0}^{2}l/{l}^{\ast }$. Here, we will assume that *l* ^*^ does not depend on the position in the material; in other words, that the material is dynamically heterogeneous, but optically homogeneous. This is a reasonable approximation for many applications in materials science; for example, spatial variations in crosslink density in a polymer material will lead to variations in the local rigidity (and therefore in the local dynamics), without causing large variations in the refractive index. To simplify the analysis further, we pass to a continuum limit and replace the summation over all paths by an integral over all path lengths *s* = *N*_*p*_ *l*:

5 $${g}_{1}({{\boldsymbol{r}}}_{{\boldsymbol{d}}},\tau )={\int }_{{s}_{{\rm{\min }}}}^{\infty }P({{\boldsymbol{r}}}_{{\boldsymbol{d}}},s)\exp (-\frac{{k}_{0}^{2}s}{3{l}^{\ast }}{\rm{\Gamma }}({{\boldsymbol{r}}}_{{\boldsymbol{d}}},\tau ,s)){\rm{d}}s$$

where *P*(***r***_***d***_, *s*) is the relative contribution of paths of length *s* to the intensity at ***r***_***d***_, *s*_min_ the minimum path length (which is of order *l* ^*^), and Γ(***r***_***d***_, *τ*, *s*) the weighted average mean square displacement of scatterers along all paths of length *s* that end at ***r***_***d***_. This can be written as

6 $${\rm{\Gamma }}({{\boldsymbol{r}}}_{{\boldsymbol{d}}},\tau ,s)=\int \langle {\rm{\Delta }}r{({\boldsymbol{r}},\tau )}^{2}\rangle \rho ({\boldsymbol{r}};{{\boldsymbol{r}}}_{{\boldsymbol{d}}},s){\rm{d}}{\bf{r}}$$

where 〈Δ*r*(***r***, *τ*)^2^〉 is the mean square displacement of particles at position ***r*** and *ρ*(***r***; ***r***_***d***_, *s*) is the normalized photon density at position ***r*** for diffusion paths of length *s* ending at ***r***_***d***_^27^. For a homogeneous medium 〈Δ*r*(***r***, *τ*)^2^〉 does not depend on ***r***, so that Γ(***r***_***d***_, *τ*, *s*) = 〈Δ*r*(*τ*)^2^〉 = 6*Dτ* with *D* the diffusion coefficient of the particles. However, for a medium with an inhomogeneous diffusivity, Γ(***r***_***d***_, *τ*, *s*) depends on what parts of the sample have been probed by the light. In this case, both the path length distribution *P*(***r***_***d***_, *s*) and the spatial density distribution of photon paths *ρ*(***r***; ***r***_***d***_, *s*) are needed to interpret the measured *g*_1_(***r***_***d***_, *τ*) in terms of the spatiotemporal dynamics in the sample.

### Photon density distribution in scattering media

To derive expressions for *P*(***r***_***d***_, *s*) and *ρ*_*s*_(***r***; ***r***_***d***_), we start by defining *G*(***r***, *n*; ***r***_**0**_,***r***_***d***_, *s*) as the probability for a random walk of length *s*, starting from a point ***r***_**0**_ on the surface of the material and ending at ***r***_***d***_, to pass through point ***r*** after a distance *n*. Since this random walk consists of two random walks, first from ***r***_**0**_ to ***r*** in *n* steps and then from ***r*** to ***r***_***d***_ in *s* − *n* steps, we can write:

7 $$G({\boldsymbol{r}},n;{{\boldsymbol{r}}}_{{\bf{0}}},{{\boldsymbol{r}}}_{{\boldsymbol{d}}},s)=\frac{G({{\boldsymbol{r}}}_{{\bf{0}}},{\boldsymbol{r}},n)G({\boldsymbol{r}},{{\boldsymbol{r}}}_{{\boldsymbol{d}}},s-n)}{G({{\boldsymbol{r}}}_{{\bf{0}}},{{\boldsymbol{r}}}_{{\boldsymbol{d}}},s)}$$

where the propagator *G*(***r***_**0**_, ***r***, *n*) gives the relative probability of paths from point ***r***_**0**_ to point ***r*** with path-length *n*. The probability of all photon paths of length *s* to a point ***r*** is

8 $$G({\boldsymbol{r}},s)=\int G({{\boldsymbol{r}}}_{{\bf{0}}},{\boldsymbol{r}},s)J({{\boldsymbol{r}}}_{{\bf{0}}})d{{\boldsymbol{r}}}_{{\bf{0}}}$$

where *J*(***r***_**0**_) is the intensity of the incident light at ***r***_**0**_. We consider here the case of homogeneous illumination, so that *J*(***r***_**0**_) is constant along the surface *z* = 0. With this, we can write

9 $$G({\boldsymbol{r}},n;{{\boldsymbol{r}}}_{{\boldsymbol{d}}},s)=\frac{G({\boldsymbol{r}},n)G({\boldsymbol{r}},{{\boldsymbol{r}}}_{{\boldsymbol{d}}},s-n)}{G({{\boldsymbol{r}}}_{{\boldsymbol{d}}},s)}$$

for the probability of all walks of length *s* ending at ***r***_***d***_ to pass through *r* after a distance *n*. The spatial density distribution of photon paths *ρ*(***r***; ***r***_***d***_, *s*) is then obtained by averaging *G*(***r***, *n*; ***r***_***d***_, *s*) over all steps^27^.

10 $$\begin{array}{rcl}\rho ({\boldsymbol{r}};{{\boldsymbol{r}}}_{{\boldsymbol{d}}},s) & = & \frac{1}{s}{\int }_{0}^{s}G({\boldsymbol{r}},n;{{\boldsymbol{r}}}_{{\boldsymbol{d}}},s)dn\\  & = & \frac{1}{sG({{\boldsymbol{r}}}_{{\boldsymbol{d}}},s)}{\int }_{0}^{s}G({\boldsymbol{r}},n)G({\boldsymbol{r}},{{\boldsymbol{r}}}_{{\boldsymbol{d}}},s-n)dn\end{array}$$

The distribution of path lengths can be calculated as:

11 $$P({{\boldsymbol{r}}}_{{\boldsymbol{d}}},s)=\frac{G({{\boldsymbol{r}}}_{{\boldsymbol{d}}},s)}{{\int }_{{s}_{{\rm{\min }}}}^{\infty }G({{\boldsymbol{r}}}_{{\boldsymbol{d}}},s)ds}$$

Within the random walk approximation that we adopt here, the propagator *G*(***r***_**0**_, ***r***, *s*) is the solution of the diffusion equation^1,2^.

12 $$\frac{\partial G}{\partial t}={D}_{{\rm{p}}}{\nabla }^{2}G$$

where the diffusion coefficient of photons in the sample can be expressed as *D*_p_ = *vl* ^*^/3, with *v* the speed of light in the material. Since the path length *s* = *vt*, we can also write this as

13 $$\frac{\partial G}{\partial s}=\frac{{l}^{\ast }}{3}{\nabla }^{2}G$$

with initial condition *G*(***r***_**0**_, ***r***, 0) = *δ*(***r*** − ***r***_**0**_). Since the incident light becomes diffuse at a depth *z* ≈ *l* ^*^, we take the beginning of the random walk in the sample to be at a distance *z*_0_ = *l* ^*^ inside the sample. The boundary condition can be specified by requiring that for *s* 0 the net flux into the sample is zero^1^. It has been shown that this is almost equivalent to forcing *G*(***r***_**0**_, ***r***, *s*) to become zero a small distance outside the sample, at the extrapolated boundary condition *z* = −*z*_*e*_, with *z*_*e*_ ≈ 0.7*l* ^*^^23,35^. The solution of the diffusion equation with this boundary condition (and constant *l* ^*^) can be obtained using the method of images^36^:

14 $$\begin{array}{c}\begin{array}{rcl}G({r}_{{\bf{0}}},r,s) & = & {(\frac{3}{4\pi s{l}^{\ast }})}^{\mathrm{3/2}}\exp (-\frac{\mathrm{3[(}x-{x}_{0}{)}^{2}+{(y-{y}_{0})}^{2}]}{4s{l}^{\ast }})\\  &  & \times \,\{\exp (-\frac{\mathrm{3(}z-{z}_{0}{)}^{2}}{4s{l}^{\ast }})-\exp (-\frac{\mathrm{3(}z+2{z}_{e}+{z}_{0}{)}^{2}}{4s{l}^{\ast }})\}\end{array}\end{array}$$

Using this result with ***r*** = ***r***_***d***_ = (*x*_*d*_, *y*_*d*_, 0), we can obtain the path length distribution from Eqs (11) and (8). Carrying out the integrations over ***r***_**0**_ and *s* gives (with *s*_min_ = 0):

15 $$P(s)=\frac{1}{4{s}^{\mathrm{3/2}}}{(\frac{3}{\pi {l}^{\ast }})}^{\mathrm{1/2}}\{{z}_{0}\exp (-\frac{3{z}_{0}^{2}}{4s{l}^{\ast }})+\mathrm{(2}{z}_{e}+{z}_{0})\exp (-\frac{\mathrm{3(2}{z}_{e}+{z}_{0}{)}^{2}}{4s{l}^{\ast }})\}$$

Since we consider homogeneous illumination and homogeneous optical properties of the material, the path length distribution does not depend on the location of the detector, so that we have dropped the dependence on ***r***_***d***_. The path length distribution *P*(*s*) has a maximum around *s* ≈ *l* ^*^, while for long paths, $s\gg {l}^{\ast }$, $P(s)\sim {s}^{-\mathrm{3/2}}$, as found previously^1^.

Next, we calculate the spatial photon density *ρ*(***r***; ***r***_***d***_, *s*) from Eq. (10). The convolution integral is most conveniently calculated by using the properties of the Laplace transform^27^:

16 $$\rho ({\boldsymbol{r}};{{\boldsymbol{r}}}_{{\boldsymbol{d}}},s)=\frac{1}{sG({{\boldsymbol{r}}}_{{\boldsymbol{d}}},s)}{ {\mathcal L} }^{-1}[ {\mathcal L} [G({\boldsymbol{r}},s)]\cdot  {\mathcal L} [G({\boldsymbol{r}},{{\boldsymbol{r}}}_{{\boldsymbol{d}}},s)]$$

where ${\mathcal L}$ denotes the Laplace transform with respect to *s*. Using the Laplace transform of Eq. (14), we find

17 $$\rho ({\boldsymbol{r}};{{\boldsymbol{r}}}_{{\boldsymbol{d}}},s)=\tfrac{3\{{R}_{1}^{-1}[\exp (-\tfrac{\mathrm{3(}{R}_{1}+|{z}_{1}{|)}^{2}}{4s{l}^{\ast }})-\exp (-\tfrac{\mathrm{3(}{R}_{1}+{z}_{2}{)}^{2}}{4s{l}^{\ast }})]-{R}_{2}^{-1}[\exp (-\tfrac{\mathrm{3(}{R}_{2}+|{z}_{1}{|)}^{2}}{4s{l}^{\ast }})-\exp (-\tfrac{3{({R}_{2}+{z}_{2})}^{2}}{4s{l}^{\ast }})]\}}{4\pi s{l}^{\ast }\{\exp (-\tfrac{3{z}_{0}^{2}}{4s{l}^{\ast }})-\exp (-\tfrac{\mathrm{3(2}{z}_{e}+{z}_{0}{)}^{2}}{4s{l}^{\ast }})\}}$$

with

$$\{\begin{array}{ccc}{R}_{1} & = & \sqrt{{(x-{x}_{d})}^{2}+{(y-{y}_{d})}^{2}+{z}^{2}}\\ {R}_{2} & = & \sqrt{{(x-{x}_{d})}^{2}+{(y-{y}_{d})}^{2}+{(z+2{z}_{e})}^{2}}\\ {z}_{1} & = & z-{z}_{0}\\ {z}_{2} & = & z+2{z}_{e}+{z}_{0}\end{array}$$

Using Eqs (5), (6), (15), and (17), we can directly relate the field correlation function measured at location ***r***_***d***_, *g*_1_(***r***_***d***_, *τ*), to the spatial distribution of particle mean-square displacements in the material, 〈Δ*r*(***r***, *τ*)^2^〉.

## Results

### Intensity distribution

**Figure 2:**
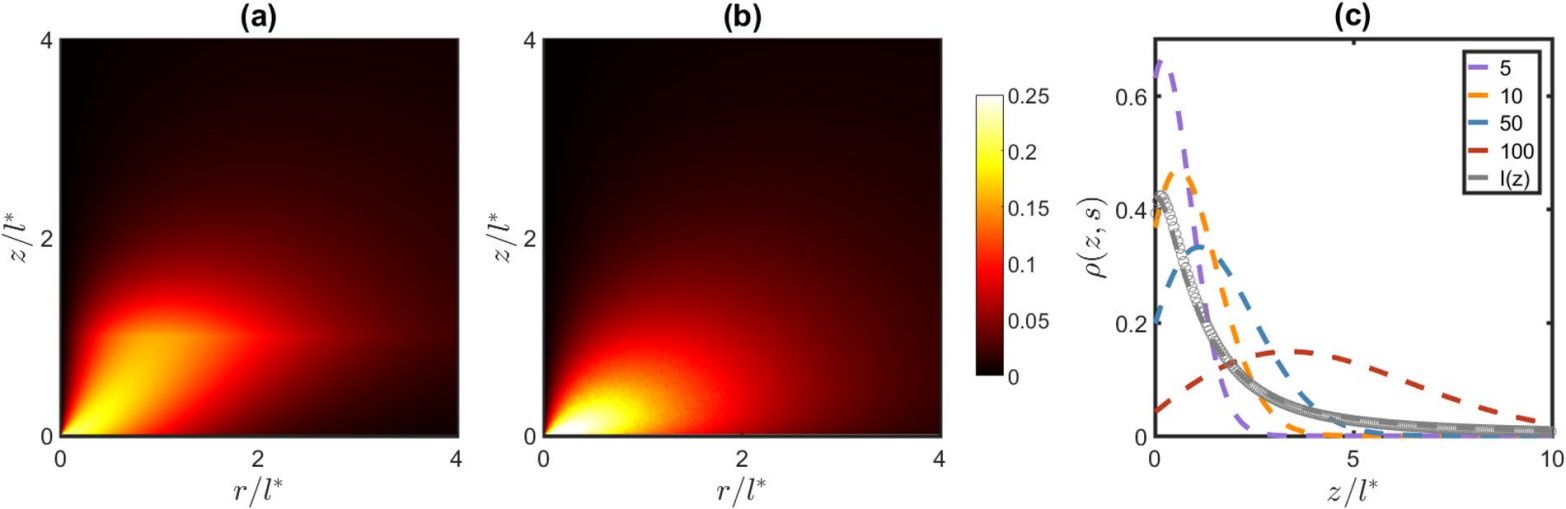
**(a)** Photon intensity distribution as a function of $r=\sqrt{{(x-{x}_{d})}^{2}+{(y-{y}_{d})}^{2}}$ and *z*, obtained from Eq. (18), with *s*_min_ = 1.3*l* ^*^. **(b)** Intensity distribution obtained with random walk simulations. **(c)** Spatial density distribution *ρ*(*z*; *s*) for photon paths of various path lengths *s*/*l* ^*^ = 5, 10, 50, and 100, and overall photon density *I* = ∫*P*(*s*)*ρ*(*z*; *s*)d*s* compared to random walk simulations (symbols).

The goal of an LSI experiment is to generate a spatially resolved image of the dynamics in the sample. The intensity of multiply scattered light that leaves the sample at *z* = 0 is recorded with a camera; from the intensity fluctuations the correlation function can be obtained for each position ***r***_***d***_ in the *z* = 0 plane. Since the photons that reach ***r***_***d***_ have taken different paths through the sample, the correlation function is a weighted average of the dynamics in the region probed by these photons. The spatial distribution of photons arriving at ***r***_***d***_ can be calculated as

18 $$I({\boldsymbol{r}};{{\boldsymbol{r}}}_{{\boldsymbol{d}}})={\int }_{{s}_{{\rm{\min }}}}^{\infty }P(s)\rho ({\boldsymbol{r}};{{\boldsymbol{r}}}_{{\boldsymbol{d}}},s){\rm{d}}s$$

and represents the relative contribution of particles in the region around ***r*** to the signal measured at ***r***_***d***_. As shown in Fig. 2a, most photons probe a region within a distance *l* ^*^ from the detector; nevertheless, a significant fraction of the photons also explores regions farther out in the sample. We have also compared our prediction for the intensity distribution with the distribution obtained from random walk simulations (Fig. 2b). For distances from the detector that are significantly larger than *l* ^*^ the agreement is very good; however, for small distances, there are clear differences, which are due to the fact that short paths are not well described by the diffusion approximation.

**Figure 3:**
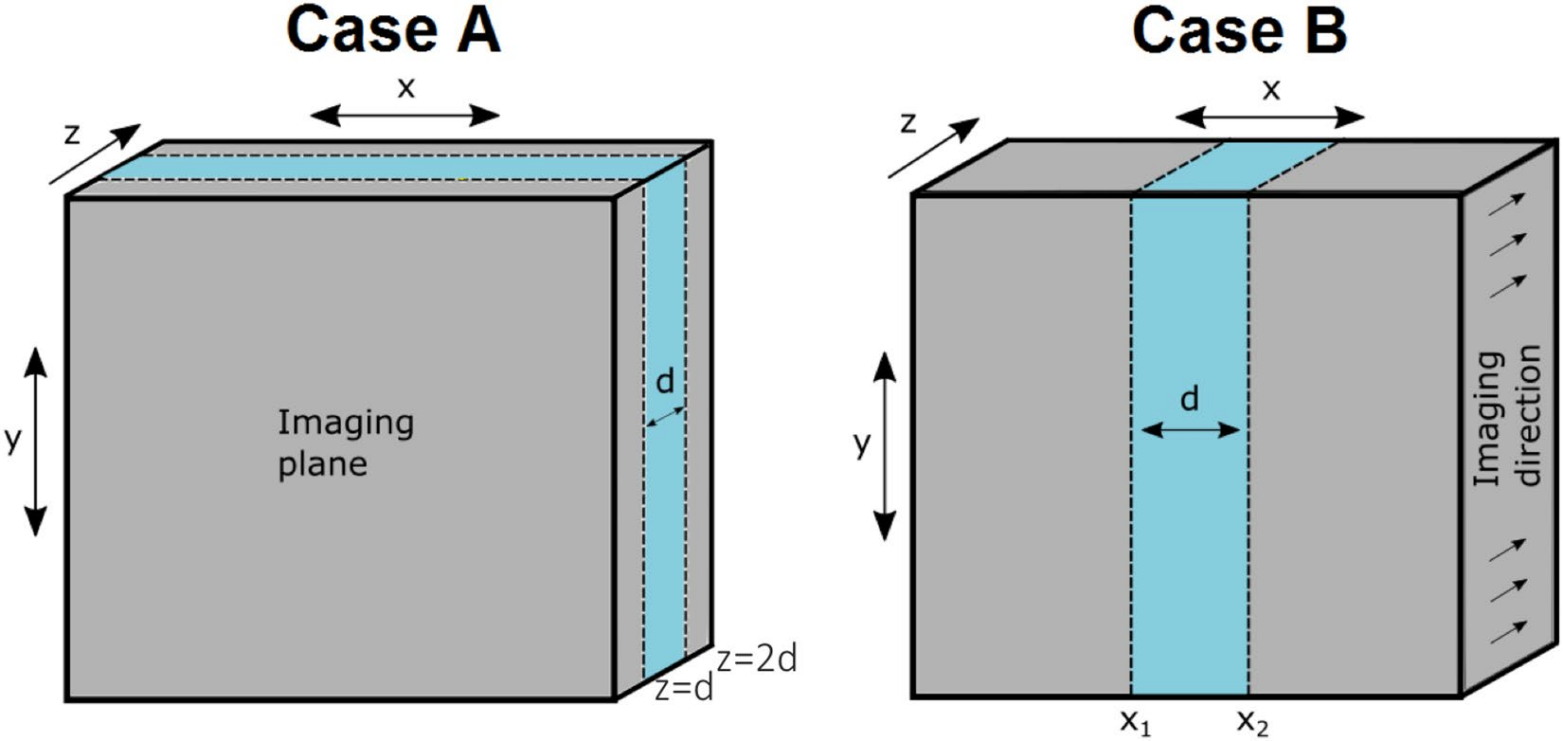
Two dynamically heterogeneous materials containing a layer of thickness *d* in which the diffusion coefficient of particles *D*_1_ differs from that in the rest of the material *D*_0_.

To investigate how this spatial distribution of photons affects the LSI signal in a dynamically heterogeneous material, we consider two different examples of materials that contain a layer of thickness *d* in which the diffusion coefficient of the particles *D*_1_ differs from that in the rest of the material *D*_0_ (Fig. 3). In case A the layer is parallel to the surface of the material, and positioned between *z* = *d* and *z* = 2*d*. This situation allows us to investigate how sensitive the LSI technique is to dynamic regions that are hidden at some depth below the surface of the material. A similar geometry has been considered previously using a slightly different approach, based on solving the correlation transport equation^31^. Here, we present an alternative expression for the autocorrelation function for this case, which we use to study the effect of *τ* and the ratio *l* ^*^/*d* on the measured autocorrelation function. We then consider case B, in which the layer is perpendicular to the surface of the material. This situation, which has not been studied quantitatively before, allows us to assess the effect of resolutional blurring in the lateral direction.

#### Dynamic heterogeneity in the *z*-direction (case A)

When the material is only heterogeneous in the *z*-direction, but not in the *xy* directions, the correlation function does not depend on the position ***r***_***d***_ of the detector and Eq. (6) can be simplified to ${\rm{\Gamma }}(\tau ,s)={\int }_{0}^{\infty }\langle \Delta r{(z,\tau )}^{2}\rangle \rho (z;s){\rm{d}}z$, where *ρ*(*z*; *s*) is the spatial distribution of photons in the *z*-direction, obtained by integrating *ρ*(***r***; ***r***_***d***_, *s*) over *x* and *y*:

19 $$\begin{array}{rcl}\rho (z;s) & = & {\int }_{-\infty }^{\infty }{\int }_{-\infty }^{\infty }\rho ({\boldsymbol{r}};{{\boldsymbol{r}}}_{{\boldsymbol{d}}},s){\rm{d}}x{\rm{d}}y\\  & = & \frac{\sqrt{3\pi }\{{\rm{erfc}}(\sqrt{\frac{\mathrm{3(|}{z}_{1}|+z{)}^{2}}{4s{l}^{\ast }}})-{\rm{erfc}}(\sqrt{\frac{\mathrm{3(|}{z}_{1}|+{z}_{3}{)}^{2}}{4s{l}^{\ast }}})-{\rm{erfc}}(\sqrt{\frac{\mathrm{3(}{z}_{2}+z{)}^{2}}{4s{l}^{\ast }}})+{\rm{erfc}}(\sqrt{\frac{\mathrm{3(}{z}_{2}+z{\mathrm{3)}}^{2}}{4s{l}^{\ast }}})\}}{2\sqrt{s{l}^{\ast }}\{\exp (-\frac{3{z}_{0}^{2}}{4s{l}^{\ast }})-\exp (-\frac{\mathrm{3(2}{z}_{e}+{z}_{0}{)}^{2}}{4s{l}^{\ast }})\}}\end{array}$$

with *z*_3_ = *z* + 2*z*_*e*_. For long paths, $s\gg {l}^{\ast }$ this can be approximated as

20 $$\rho (z;s)\approx \frac{\mathrm{6(}z+{z}_{e})}{s{l}^{\ast }}\,\exp (-\frac{\mathrm{3(}z+{z}_{e}{)}^{2}}{s{l}^{\ast }})$$

As shown in Fig. 2c, the photon density for diffusion paths of a given length *s* has a maximum around a depth *z*_max_ ≈ (*sl* ^*^/6)^1/2^. Particles at this depth contribute most to the decorrelation of these paths. The total photon density, obtained by integrating over all path lengths, *I*(*z*) = ∫*P*(*s*)*ρ*(*z*; *s*)d*s*, is highest close to the surface of the material and decays as $I(z)\sim {z}^{-2}$ for larger *z*.

**Figure 4:**
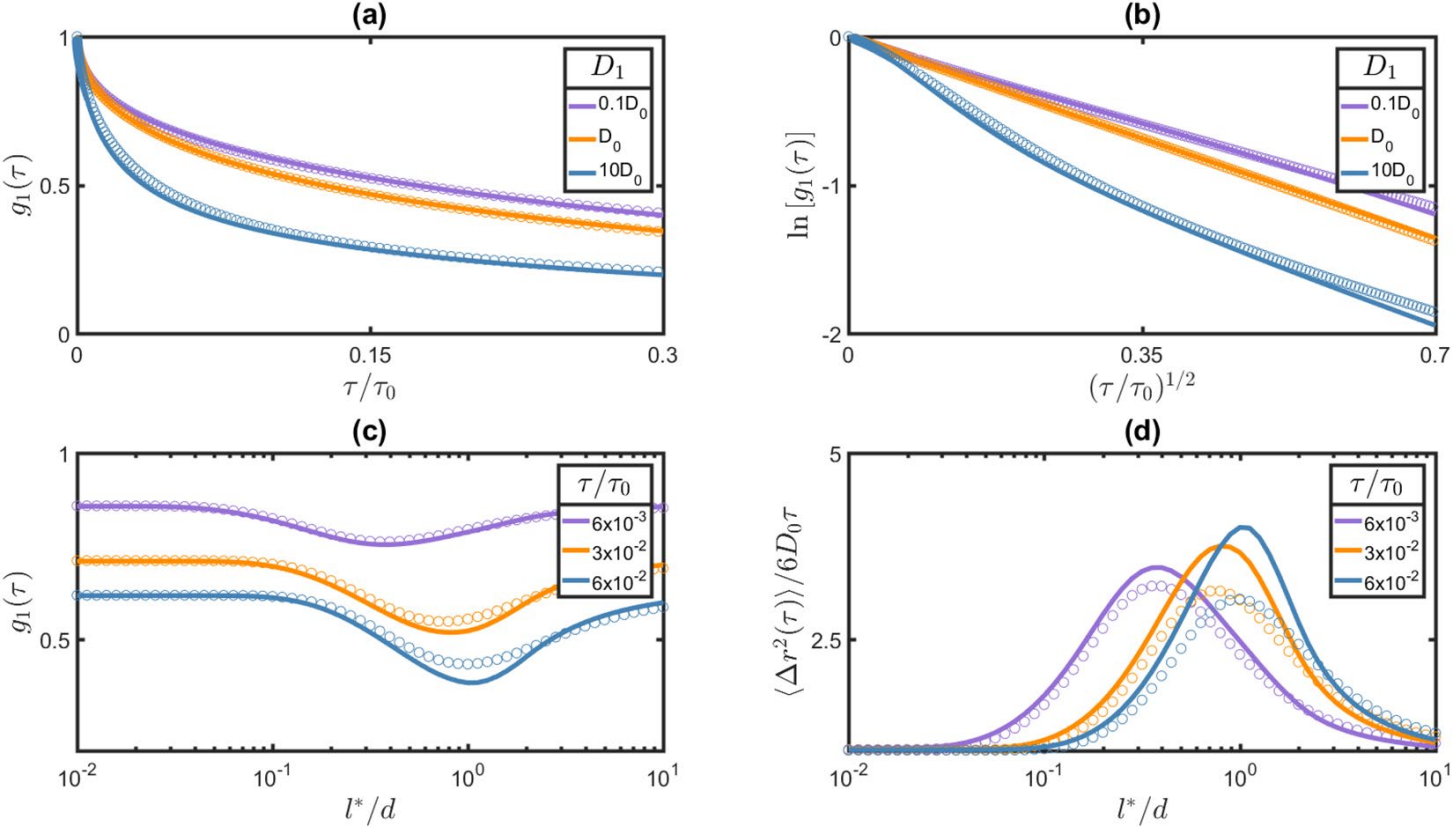
LSI results for samples with a layer positioned between *z* = *d* and *z* = 2*d* in which the diffusion coefficient *D*_1_ differs from that in the rest of the material *D*_0_ (case A in Fig. 3). Lines represent theoretical results and symbols random walk simulations. **(a)** Field correlation functions *g*_1_(*τ*) as a function of the renormalized time *τ*/*τ*_0_ with ${\tau }_{0}=(6{k}_{0}^{2}{D}_{0}{)}^{-1}$, for *l* ^*^ = 2*d*/3 and different ratios *D*_1_/*D*_0_. **(b)** ln[*g*_1_(*τ*)] versus *τ* ^1/2^ for the same parameters. **(c)** Correlation function *g*_1_(*τ*) for *τ*/*τ*_0_ = 6 × 10^−3^, 3 × 10^−2^, and 6 × 10^−2^ and *D*_1_ = 10*D*_0_ as a function of *l* ^*^/*d*. **(d)** Apparent mean square displacements obtained from the *g*_1_(*τ*) values in (**c**) using Eq. (21).

In Fig. 4a, we compare the field correlation function *g*_1_(*τ*) for a homogeneous sample (*D*_1_ = *D*_0_) with that in a sample in which the diffusion coefficient in the layer *D*_1_ is either a factor of 10 higher or lower than that in the rest of the material *D*_0_, for *l* ^*^ = 2*d*/3. Clearly, the decorrelation is slowed down by a slowly diffusing layer, while it is accelerated by a rapidly diffusing layer. For a homogeneous sample, the correlation function is given by^1,4^

21 $${g}_{1}(\tau )\approx \exp (-\gamma {k}_{0}\sqrt{\langle {\rm{\Delta }}r{(\tau )}^{2}\rangle })=\exp (-\gamma \sqrt{\tau /{\tau }_{0}\rangle })$$

with *γ* = (*z*_0_ + *z*_*e*_)/*l* ^*^ ≈ 1.7 and ${\tau }_{0}={\mathrm{(6}{k}_{0}^{2}{D}_{0})}^{-1}$ the time for particles in the medium to diffuse a distance ${k}_{0}^{-1}$. It follows that a plot of *ln*[*g*_1_(*τ*)] versus *τ* ^1/2^ should give a straight line with slope $-\gamma /\sqrt{{\tau }_{0}}$. This is indeed what we find (orange data in Fig. 4b). Here, the short correlation times, corresponding to fast decorrelation, originate from the long paths with many scattering events, while the long correlation times originate from the short paths with only few scattering events. For a material with a layer of different diffusivity (*D*_1_ ≠ *D*_0_), we find that the initial decay follows that of the homogeneous sample. This initial decorrelation is due to the very long paths, $s\gg {d}^{2}/{l}^{\ast }$ that mostly sample the region *z* > 2*d*. For intermediate correlation times, we find a faster decay for *D*_1_ *D*_0_ and a slower decay for *D*_1_ < *D*_0_, with a slope in Fig. 4b that differs roughly by a factor $\sqrt{{D}_{1}/{D}_{0}}$ . This different decay is due to the paths with a length on the order of 6*d* ^2^/*l* ^*^ that sample the diffusive layer. At longer correlation times, where we mainly see the short photon paths that sample the region *z* < *d*, the slope returns to that of the homogeneous sample.

The extent to which photons probe different depths in the sample depends on the transport mean free path *l* ^*^ (and thus on the wavelength). We therefore expect the measured dynamics in a material with a *z*-dependent diffusion coefficient to depend strongly on the value of *l* ^*^. To investigate this, we calculate the correlation function for the case with *D*_1_ = 10*D*_0_ for different ratios *l* ^*^/*d*. The largest effect of the diffusive layer is observed when *l* ^*^ is on the order of the layer depth *d* (Fig. 4c). When *l* ^*^ is much smaller, the photons do not penetrate deep enough in the sample to reach the layer, while for much larger *l* ^*^ the photons travel much deeper in the sample and only spend a small fraction of the time in the layer. We also find that for longer correlation times *τ*, the optimum *l* ^*^ for which the effect of the diffusive layer is highest gradually shifts to higher values. Again, the reason for this is that longer decay times correspond to paths with fewer scattering events, so that a larger *l* ^*^ is needed for these paths to reach the diffusive layer. These findings are in agreement with previously obtained results that showed that the correlation time at which the largest effect of the diffusive layer is observed decreases with increasing depth *d* of the layer^31^. Usually, in the analysis of LSI data the correlation functions *g*_1_(***r***_***d***_, *τ*) are converted to mean square displacements using Eq. (21), implicitly assuming that the material is homogeneous in the *z*-direction. Doing this, one obtains an apparent mean square displacement, which is a weighted average over the *z*-range probed by the photons. As shown in Fig. 4d, this apparent diffusion coefficient lies between *D*_0_ and *D*_1_, depending on the ratio *l* ^*^/*d* and the correlation time *τ*, and reaches at most 30 to 40 percent of *D*_1_.

#### Dynamic heterogeneity in the lateral direction (case B)

When the material is homogeneous in the *z*-direction, but inhomogeneous in the *xy*-plane, the measured correlation function depends on the position of the detector and is given by the photon density *ρ*(*x* − *x*_*d*_, *y* − *y*_*d*_; *s*), which is obtained from *ρ*(***r***; ***r***_***d***_, *s*) by integrating over *z*:

22 $$\begin{array}{rcl}\rho (x-{x}_{d},y-{y}_{d};s) & = & {\int }_{0}^{\infty }\rho ({\boldsymbol{r}};{{\boldsymbol{r}}}_{{\boldsymbol{d}}},s){\rm{d}}z\\  & = & \frac{3}{4\pi s{l}^{\ast }}{E}_{1}(\frac{3[{(x-{x}_{d})}^{2}+{(y-{y}_{d})}^{2}]}{4s{l}^{\ast }})\end{array}$$

with ${E}_{1}(x)={\int }_{x}^{\infty }({e}^{-t}/t){\rm{d}}t$ the exponential integral function. The lateral spreading of photons can be quantified by the second moment of this distribution,

23 $$\langle {(r-{r}_{d})}^{2}\rangle ={\int }_{0}^{\infty }2\pi {r}^{3}\rho (r-{r}_{d},s){\rm{d}}r=\frac{2s{l}^{\ast }}{3}$$

with *r* − *r*_*d*_ = ((*x* − *x*_*d*_)^2^ + (*y* − *y*_*d*_)^2^)^1/2^. If the material is heterogeneous only in one direction (as in Fig. 3B), we can also integrate over *y*, to obtain

24 $$\begin{array}{rcl}\rho (x-{x}_{d},s) & = & {\int }_{0}^{\infty }{\int }_{-\infty }^{\infty }\rho ({\boldsymbol{r}};{{\boldsymbol{r}}}_{{\boldsymbol{d}}},s){\rm{d}}y{\rm{d}}z\\  & = & \sqrt{\frac{3}{\pi s{l}^{\ast }}}\exp (-\frac{\mathrm{3(}x-{x}_{d}{)}^{2}}{4s{l}^{\ast }})-\frac{\mathrm{3|}x-{x}_{d}|}{2s{l}^{\ast }}{\rm{erfc}}(\sqrt{\frac{\mathrm{3(}x-{x}_{d}{)}^{2}}{4s{l}^{\ast }}})\end{array}$$

which has a second moment 〈(*x* − *x*_*d*_)^2^〉 = *sl* ^*^/3.

**Figure 5:**
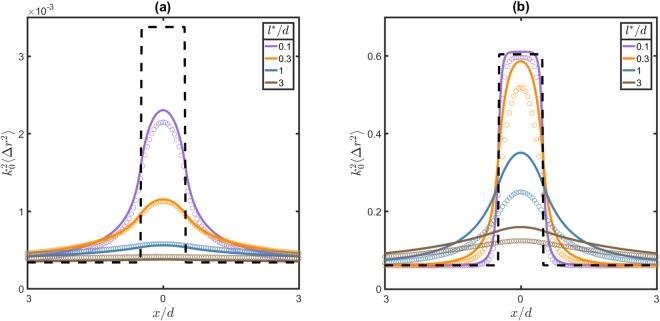
Apparent mean square displacement for samples with a layer of higher diffusivity with *D*_1_ = 10*D*_0_, placed between *x* = −*d*/2 and *x* = *d*/2 (case B in Fig. 3), for *l* ^*^/*d* = 0.1, 0.3, 1, and 3, and for *τ*/*τ*_0_ = 3 × 10^−4^ **(a)** and 6 × 10^−2^ **(b)**. Lines represent theoretical results and symbols random walk simulations. The dashed black line represents the real mean square displacements.

To see how this lateral spreading of photons influences the resolution of LSI in dynamically heterogeneous materials, we consider a material that contains a layer of width *d* perpendicular to the imaging plane in which the diffusion coefficient is 10 times higher than that in the rest of the material (case B in Fig. 3). We calculate the correlation function as a function of the detector position *x*_*d*_ using Eqs (15) and (24), and convert this into an apparent mean square displacement using Eq. (21). Figure 5 shows the apparent mean square displacements for two different correlation times and various *l* ^*^. It is clear that the actual mean square displacement is more accurately followed for small *l* ^*^ values. As expected, when *l* ^*^ > *d*, the photons explore a region that is much larger than the width of the layer, leading to smoothing of the profile and a strongly reduced imaging contrast. However, even when *l* ^*^ is ten times smaller than *d*, the smoothing is still very significant for short correlation times (Fig. 5a), and the apparent mean square displacement measured in the layer is almost two times smaller than the actual mean square displacement. For longer correlation times, the blurring is significantly smaller (Fig. 5b); this is explained by the fact that these longer correlation times correspond to shorter paths with fewer scattering events, which sample a smaller region of the sample (Eq. (23)), therefore causing less blurring.

## Discussion

We have obtained theoretical expressions for the path length distribution and spatial density distribution of photons in a strongly scattering, semi-infinite medium under plane wave illumination. These expressions allowed us to calculate the dynamic correlation function that would be measured in a backscatter LSI experiment for dynamically heterogeneous materials. We have applied these results to two simple geometries of a layered medium with different diffusivities, but our expressions can be used for any spatial distribution of diffusion coefficients. Moreover, the effects of directional flows with spatially varying shear rates $\dot{\gamma }({\boldsymbol{r}})$ can be included straightforwardly, by adding a term $\tfrac{1}{5}{(\tau {l}^{\ast })}^{2}\int \dot{\gamma }{({\boldsymbol{r}})}^{2}\rho ({\boldsymbol{r}};{{\boldsymbol{r}}}_{{\boldsymbol{d}}},s)d{\boldsymbol{r}}$ to Eq. (6), which accounts for the decorrelation due to spatially inhomogeneous flows^5,27^.

Our model makes use of the diffusion approximation for describing the transport of photons in the medium, which is known to be accurate for sufficiently long paths, $s\gg {l}^{\ast }$. It is not obvious that this condition holds for backscattering LSI, in which short paths contribute significantly to the signal; for example, according to Eq. (15) the path length distribution has a maximum for *s* ≈ *l* ^*^. This is aggravated by our neglect of polarization effects, which are important for short photon paths for which depolarization does not yet occur^33^. To suppress the contribution of these short paths, we have assumed that the random walk starts at a depth *z* ≈ *l* ^*^ and we have imposed a minimum path length *s*_min_ ≈ *l* ^*^. Likewise, in the simulations, we only considered photons that were scattered at least twice. This corresponds to the experimental situation, where singly scattered photons are usually excluded from the analysis by using a polarization filter. With these conditions, we find remarkably good agreement between the theoretical results (with *s*_min_ = 1.3*l* ^*^) and the random walk simulations (Figs 2, 4, and 5). The agreement is especially good for short correlation times *τ*, which correspond to the long diffusion paths, with many scattering events. For longer *τ*, corresponding to shorter paths, the diffusion approximation loses its validity, leading to larger differences between the theoretical results and the simulations (see e.g. Figs 4c,d and 5b). Nevertheless, even in this case there is still very good qualitative agreement.

Our results show that the interpretation of LSI measurements in dynamically heterogeneous samples should be done with care. The apparent diffusion coefficient measured at a particular position can depend strongly on the transport free path *l* ^*^ and the correlation time *τ*. For larger *l* ^*^ and for shorter *τ*, the decorrelation is due to longer photon paths, which probe regions that are deeper inside the material, while for small *l* ^*^ and large *τ* the decorrelation is due to short paths that probe the regions near the surface (Fig. 4). For the same reason, the resolution of LSI in the lateral direction is smaller for large *l* ^*^ and small *τ* due to the spatial averaging that is inherent for the longer photon paths. For short correlation times, significant spatial blurring occurs even on length scales that are more than ten times larger than *l* ^*^ (Fig. 5). The outcome of an LSI measurement in a dynamically heterogeneous material thus depends strongly on the value of *l* ^*^ and *τ*. For a reliable interpretation, it is therefore recommended to perform measurements for a range of correlation times, and preferably also for different *l* ^*^. We believe that our theory will be useful for analyzing the results of such measurements and for estimating the effects of spatial averaging, and will therefore contribute to an improved accuracy of LSI measurements.

## Methods: random walk simulations

To validate the expressions obtained for the photon density distribution and the autocorrelation function, we use random walk simulations^37^. We collect statistics for 10^6^ photons, which are launched one at a time in the +*z*-direction at *z* = 0, and allowed to perform a random walk until they leave the sample again at *z* = 0. The step length is sampled from a Poisson distribution with mean *l* ^*^, and we assume isotropic scattering, so that the direction of each step is random. For each step we calculate the transfer wave vector ***q***_***i***_, and we record the accumulated phase shift $\langle {\rm{\Delta }}{\varphi }^{2}(\tau )\rangle ={\sum }_{i}{q}_{i}^{2}\langle {\rm{\Delta }}r{({{\bf{r}}}_{{\bf{i}}},\tau )}^{2}\rangle$, with 〈Δ*r*(***r***_***i***_, *τ*)^2^〉 = 6*D*(***r***_***i***_)*τ* the mean square displacement of particles at the location of scattering event *i*. The field correlation function *g*_1_(*τ*) is obtained from this by averaging $\exp (-\tfrac{1}{2}\langle {\rm{\Delta }}{\varphi }^{2}(\tau )\rangle )$ over all random walks^37^. Since we are dealing with multiple scattering, we only consider trajectories with at least 2 scattering events.

## References

[1] Pine, D., Weitz, D., Chaikin, P. & Herbolzheimer, E. Diffusing wave spectroscopy. *Phys.Rev.Lett*. 1134–1137 (1988). 10.1103/PhysRevLett.60.1134 10037950

[2] Maret G, Wolf P (1987). Multiple light scattering from disordered media - the effect of brownian motion of scatterers. Z. Phys. B - Condensed Matter.

[3] Viasnoff V, Lequeux F, Pine D (2002). Multispeckle diffusing-wave spectroscopy: A tool to study slow relaxation and time-dependent dynamics. Rev. Sci. Instrum..

[4] Pine D, Weitz D, Zhu J, Herbolzheimer E (1990). Diffusing wave spectroscopy: dynamic light scattering in the multiple scattering limit. J.Phys.France.

[5] Wu X, Pine D, Chaikin P, Huang J, Weitz D (1990). Diffusing-wave spectroscopy in a shear flow. J. Opt. Soc. Am. B.

[6] Bicout D, Akkermans E, Maynard R (1991). Dynamical correlations for multiple light scattering in laminar flow. J. Phys. I.

[7] Mason T, Weitz D (1995). Optical measurements of frequency-dependent linear viscoelastic moduli of complex fluids. Phys.Rev.Lett..

[8] Boas D, Campbell L, Yodh A (1995). Scattering and imaging with diffusing temporal field correlations. Phys. Rev. Lett..

[9] Boas D, Yodh A (1997). Spatially varying dynamical properties of turbid media probed with diffusing temporal light correlation. Journal of the Optical Society of America A.

[10] Heckmeier M, Skipetrov S, Maret G, Maynard R (1997). Imaging of dynamic heterogeneities in multiple-scattering media. J. Opt. Soc. Am. B.

[11] Boas DA, Dunn AK (2010). Laser speckle contrast imaging in biomedical optics. J Biomed Opt..

[12] Nadkarni SK (2005). Characterization of atherosclerotic plaques by laser speckle imaging. Vascular Medicine.

[13] Regan C (2015). Fiber-based laser speckle imaging for the detection of pulsatile flow. Lasers in Surgery and Medicine.

[14] Nemati M (2015). Application of full field optical studies for pulsatile flow in a carotid artery phantom. Biomedical Optics Express.

[15] Basak K, Dey G, Mahadevappa M, Mandal M, Sheet D (2016). Learning of speckle statistics for *in vivo* and noninvasive characterization of cutaneous wound regions using laser speckle contrast imaging. Microvascular Research.

[16] Zakharov P (2009). Dynamic laser speckle imaging of cerebral blood flow. Optics express.

[17] van der Kooij, H. M., Fokkink, R., van der Gucht, J. & Sprakel, J. Quantitative imaging of heterogeneous dynamics in drying and aging paints. *Scientific Reports* **6** (2016). 10.1038/srep34383 PMC5041151 27682840

[18] van der Kooij H, Susa A, Garcia S, van der Zwaag S, Sprakel J (2017). Imaging the molecular motions of autonomous repair in a self-healing polymer. Adv. Mater..

[19] Amon A, Mikhailovskaya A, Crassous J (2017). Spatially-resolved measurements of micro-deformations in granular materials using dws. Rev. Sci. Instr..

[20] Erpelding M, Amon A, Crassous J (2008). Diffusive wave spectroscopy applied to the spatially resolved deformation of a solid. Phys. Rev. E.

[21] Kashany, Z. H. *et al*. Laser speckle micro rheology for micro-mechanical mapping of bio-materials. *Proc. SPIE 9707, Dynamics and Fluctuations in Biomedical Photonics XIII, 970702* **970702** (2016).

[22] Alvarado FAP, Hussein MA, Becker T (2015). Laser speckle spectroscopy image analysis for high pressure and high temperature treatment discrimination on ldpe, hdpe bopp, bopa and pet polymer layers used for food packaging. Journal of Food Process Engineering.

[23] Ishimaru, A. *Wave propagation and scattering in random media* (Academic Press, New York., 1978).

[24] Bianco SD, Martelli F, Zaccanti G (2002). Penetration depth of light re-emitted by a diffusive medium: theoretical and experimental investigation. Physics in Medicine and Biology.

[25] Martelli F (2016). There’s plenty of light at the bottom: statistics of photon penetration depth in random media. Scientific Reports.

[26] Bicout DJ, Weiss GH (1998). A measure of photon penetration into tissue in diffusion models. Optics Communications.

[27] Bicout D, Maynard R (1993). Diffusing wave spectroscopy in inhomogeneous flows. Physica A: Statistical Mechanics and its Applications.

[28] Zakharov P, Völker A, Buck A (2006). B, B. W. & Scheffold, F. Quantitative modeling of laser speckle imaging. Opt. Lett..

[29] Scheffold, F. Principles and fundamentals of optical imaging. In Helmchen, F. & Weber, B. (eds) *Optical Imaging of Neocortical Dynamics* (Springer, 2014).

[30] Zakharov P, Scheffold F (2010). Monitoring spatially heterogenous dynamics in a drying colloidal thin film. Soft Materials.

[31] Skipetrov S, Maynard R (1996). Dynamic multiple scattering of light in multilayer turbid media. Physics Lett. A.

[32] Scheffold F, Skipetrov S, Romer S, Schurtenberger P (2001). Diffusing-wave spectroscopy of nonergodic media. Phys. Rev. E.

[33] Rojas-Ochoa L, Lacoste D, Lenke R, Schurtenberger P, Scheffold F (2004). Depolarization of backscattered linearly polarized light. J. Opt. Soc. Am. A.

[34] Irimpan L, Dann V, Krishnan B (2008). Backscattering of laser light from colloidal silica. Laser Phys..

[35] van der Mark MB, van Albada MP, Lagendijk A (1988). Light scattering in strongly scattering media: Multiple scattering and weak localization. Phys. Rev. B.

[36] Contini D, Martelli F, Zaccanti G (1997). Photon migration through a turbid slab described by a model based on diffusion approximation. i. theory. Appl. Opt..

[37] Durian D (1995). Accuracy of diffusing-wave spectroscopy theories. Phys. Rev. E.

